# Effects of Desert Olive Tree Pearls Containing High Hydroxytyrosol Concentrations on the Cognitive Functions of Middle-Aged and Older Adults

**DOI:** 10.3390/nu15143234

**Published:** 2023-07-21

**Authors:** Jieun Yoon, Kazunori Sasaki, Iku Nishimura, Haruna Hashimoto, Tomohiro Okura, Hiroko Isoda

**Affiliations:** 1 Institute of Health and Sport Sciences, University of Tsukuba, 1-1-1 Tennodai, Tsukuba 305-8572, Japan; okura.tomohiro.gp@u.tsukuba.ac.jp; 2 Open Innovation Laboratory for Food and Medicinal Resource Engineering, National Institute of Advanced Industrial Science and Technology (AIST), 1-1-1 Tennodai, Tsukuba 305-8572, Japan; sasaki-kazu@aist.go.jp; 3 R&D Center for Tailor-Made QOL, University of Tsukuba, 1-2 Kasuga, Tsukuba 305-0821, Japan; hashimoto.haruna.gb@un.tsukuba.ac.jp; 4 Doctor Program in Physical Education Health and Sport Sciences, University of Tsukuba, 1-1-1 Tennodai, Tsukuba 305-8572, Japan; s2330498@u.tsukuba.ac.jp; 5 Institute of Life and Environmental Sciences, University of Tsukuba, 1-1-1 Tennodai, Tsukuba 305-8572, Japan

**Keywords:** olive oil, desert olive tree pearls, polyphenols, hydroxytyrosol, cognitive function, brain activity

## Abstract

The Japanese population has the world’s longest life expectancy but faces the challenge of extending the healthy life expectancy without developing chronic diseases. Therefore, the effectiveness of products derived from olives used in the Mediterranean diet as a potential dietary solution has attracted attention. This study examined the effects of desert olive tree pearls (DOTPs), which contain 162 times more polyphenol hydroxytyrosol than olive oil, on the cognitive function of middle-aged and older adults using the Cognitrax test. Participants (aged 51–82 years) were assigned to the DOTP intake (*n* = 36) or placebo group (*n* = 36) in a randomized, double-blind, placebo-controlled, parallel-group study. The participants received 3 g of DOTPs or placebo in olive oil twice daily for 12 weeks. Among cognitive domains, complex attention had a significant time × group interaction effect (*p* = 0.049) between the DOTP and placebo groups. The simple main effect for this item was significantly different (*p* < 0.001 and *p* = 0.572, respectively). Time effects were significant (*p* < 0.05) for the psychomotor speed, reaction time, cognitive flexibility, processing speed, and executive function domains. Therefore, DOTPs have the potential to alleviate cognitive problems faced by middle-aged and older adults in Japan.

## 1. Introduction

Japan is transforming into a super-aging society as the Japanese people have a long life expectancy, with an average life span of 84.3 years [1]. In 2000, the Long-Term Care Insurance Law was enacted to address various problems faced by older adults [1]. However, it has become a major burden both individually and socially. Hence, the Japanese government is taking measures to extend the healthy life expectancy or maintain the health and well-being of older adults. Maintaining and improving the cognitive function of older adults are challenging, and the increase in incidence of age-related dementia has become a serious problem. Moreover, the high cost and uncertain efficacy of anti-dementia drugs make it difficult for the older adults to take any other special preventive measures. Although anti-dementia medications are effective in older adults with Alzheimer’s disease (AD), these medications have a variety of side-effects. In Japan, alternatives to improve the cognitive function of the older adults other than medications remain unexplored. Older adults at risk of chronic diseases are often prescribed multiple medications, which puts them at a high risk of adverse drug reactions and possible side-effects. This situation thus warrants a regime that is a combination of diet, natural supplements, and exercise, instead of medications.

Recently, nutrition counseling and diet adjustment have shown positive results in extending the healthy lifespan and preventing various diseases, such as cancer, obesity, and AD [2]. Following a specific diet, particularly the Mediterranean diet (MedDiet), can reduce the risk of developing certain diseases, and certain components of the diet can exert therapeutic effects [3,4]. Japanese older adults consume Mediterranean foods and related supplements to maintain or improve their cognitive and health functions. Olive oil products are among the most representative components of the MedDiet. Olive oil contains hydroxytyrosol (HYD), which has been associated with improved composite measures of cognitive function in older adults [5]. Desert olive tree pearls (DOTPs), which are rich in HYD, have several health benefits, including antioxidant, anti-inflammatory, anti-atherogenic, and anti-thrombotic effects [6]. HYD, which can be extracted from olive leaves and fruits, is stable in its free state and readily penetrates human tissues [7]. An animal study reported that DOTPs reduce Aβ levels in the brain, improves learning and memory, and enhances the activity of brain neurotransmitters in aged mice [6]. However, the effects of DOTPs on the cognitive function of older adults remain unclear to date.

Therefore, this study aimed to investigate the effects of HYD-rich DOTPs on the cognitive function of older adults. In this double-blind, randomized, placebo-controlled trial, we compared changes in the cognitive function (memory, attention, reaction time, and executive function) of middle-aged and older adults who received DOTPs and placebo for 12 weeks.

## 2. Materials and Methods

### 2.1. Ethical Statement and Participants

**Figure 1:**
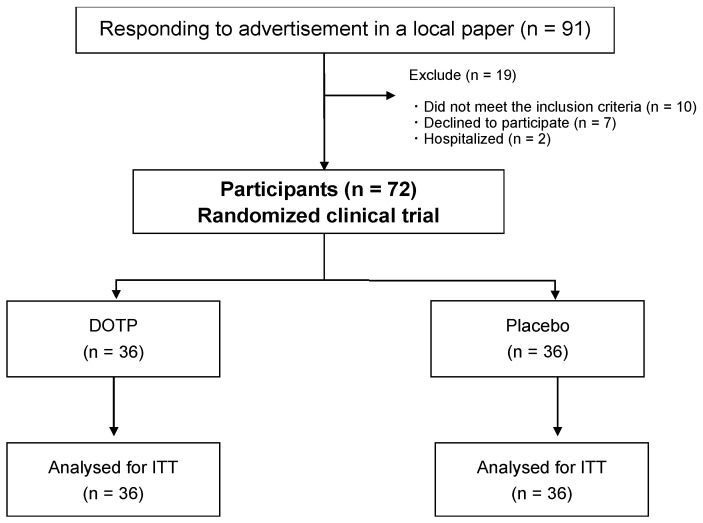
Randomized controlled trial flowchart of this study (DOTP: desert olive tree pearl group, PLAG: placebo group, ITT: intention to treat).

This randomized, double-blind, placebo-controlled, parallel-group study was approved by the ethics committee of the University of Tsukuba (reference no. Tai 021-120) and registered at the University Hospital Medical Information Network Center (UMIN no. R00005196). To ensure the reliability of the double-blind trial, we entrusted the recruitment and data management of the participants to a business consignment agency (e-sport corporation, Tsukuba, Japan). Middle-aged and older adults residing in Tsukuba City, Japan, were recruited over a one-month period through regional information magazines (Joyo Living Co., Ltd., Tsukuba, Japan). A screening survey was conducted via telephone interviews using self-reported, general health questionnaires. The inclusion criteria were as follows: (1) middle-aged and older adults and (2) people who can independently visit the experimental facility. Participants were excluded if they (1) had paralysis, (2) had an allergic reaction to the ingredients of the test food, (3) were participating in other clinical studies or had participated within three months, and (4) were deemed by the principal investigator to be inappropriate as participants of this study. A total of 91 middle-aged and older adults were enrolled in this study; however, 10 of these individuals were excluded in accordance with the criteria, 7 declined to participate, and 2 were hospitalized (Figure 1). Thus, 72 individuals participated in this study. The study was conducted using methodologies that complied with all ethical standards enunciated in the Declaration of Helsinki and its amendments [8]. Eligible participants were fully informed of the study objectives, design, inclusion and exclusion criteria, intervention of supplements, assessments, insurance compensation for injury, withdrawal of consent, and privacy protection. All participants provided written informed consent. This 12-week study was conducted between 4 April 2022 and 26 June 2022.

### 2.2. Desert Olive Tree Pearls (DOTPs) and Placebo Samples

**Figure 2:**
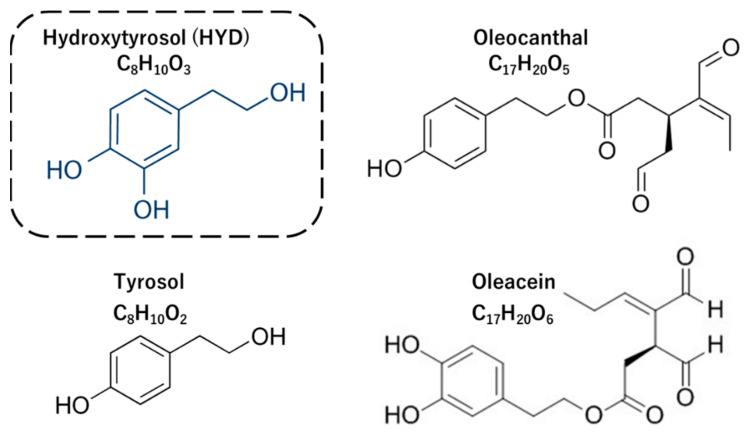
Structures of major polyphenols in desert olive tree pearls.

The DOTPs used in this study were obtained from the Atlas Olive Oils Company in Morocco. DOTPs were prepared from the leaves, baby leaves, and fruits of olive trees using cold agitation and high-pressure mechanical manufacturing methods and contain various polyphenols, such as HYD, tyrosol, oleocanal, and oleacein (analyzed using HPLC by Laboratoire de Biotechnologie, Ryad, Morocco) (Table 1 and Figure 2). As shown in Table 1, the DOTPs used in this study contained a higher amount of HYD (16.2 mg/g) than the other polyphenols. The placebo contained cornstarch instead of DOTPs and was prepared to match the shape and color of DOTPs; both were gel-type granules dipped in olive oil.

The DOTP group consumed 3 g of DOTPs with HYD twice a day (for breakfast and dinner) and the placebo group consumed a non-HYD DOTP placebo. The participants were asked to consume a uniform diet during the study and maintain a daily intake diary to keep track of the diet.

### 2.3. Anthropometric Assessment

Anthropometric indices, including height and weight, were measured at the beginning and end of the 12-week intervention period. The body mass index (BMI) was calculated as weight divided by height squared.

### 2.4. Evaluation of Cognitive Function

The Cognitrax test (Health Solution, Inc., Tokyo, Japan) was used to assess the cognitive function of the participants after the intervention. It consists of a computerized test battery that evaluates multiple cognitive domains, including composite memory, verbal memory, visual memory, processing speed, psychomotor speed, executive function, reaction time, complex attention, simple attention, cognitive flexibility, and motor speed. The Verbal Memory (VBM) and Visual Memory (VIM) tests are adaptations of the Rey Auditory Verbal Learning and the Rey Visual Design Learning Tests. The total number of correct answers on VBM and VIM becomes the memory aggregate score or the memory area score. Psychomotor speed is obtained from the sum of the left and right finger taps of the Finger Tapping Test (FTT) and the sum of the number of correct answers to the Symbol Digit Coding (SDC). FTT is one of the core tests of the Halstead–Reitan Battery, whereas SDC is based on the Symbol 3 Digit Modalities Test, a variant of the Wechsler digit symbol substitution test. Within the Central Nervous System Vital Signs (CNSVSs), the Stroop Test (ST) averages the two complex reaction time scores from simple and complex reaction times to obtain a score in the “reaction time” domain. From this domain, the “information processing speed” is also obtained. The Shifting Attention Test (SAT) measures a subject’s ability to respond quickly and accurately from one instruction to the next. The Cognitive Adaptability domain score is the number of correct answers on the SAT minus the total number of incorrect answers on the SAT and ST. The Continuous Performance Test (CPT) measures sustained attention. The domain score for total attention is the sum of the number of incorrect responses on the CPT, SAT, and ST. We used stimuli from the “stores” of words and shapes in the test (VBM, VIM, SDC). Reaction times were recorded with millisecond precision in the test (VBM, VIM, FTT, ST, SAT, CPT).

Cognitrax scores have been standardized according to results from a large population of participants aged 7–90 years [9].

### 2.5. Statistical Analysis

We analyzed all available data by treatment allocation, following the principles of intention to treat.

All statistical analyses were performed using SPSS (version 26, IBM, Armonk, NY, USA), with a significance set at *p* < 0.05. All data were checked for normality of distribution using the Shapiro–Wilk test.

We used baseline characteristics as the mean and compared them to the standard deviation or the frequency count between the groups using Student’s *t*-test and χ^2^ test.

Repeated-measures ANCOVA with years of education as a covariate was used to determine whether differences in all outcome measures existed between values (two levels for the time factor: pre- and post-trial) for each group (group factor: DOTP and placebo groups). When the interaction was significant, we examined the effect of time on each group. When the interaction was not significant, we tested the main effects of time and group. We calculated outcome measures of pre- and post-trials with a 95% confidence interval. The effect sizes (Cohen’s *d*) of the pre- and post-trial data were determined using the average change, excluding the pre-test standard deviation.

## 3. Results

### 3.1. Participants

A total of 72 participants were randomly assigned to the DOTP (*n* = 36) or placebo group (*n* = 36) (Figure 1) for a 12-week trial. No significant differences in demographic and clinical variables were observed between the two groups at baseline (Table 2), and the intake rate of all groups was 97.2% (97.5% and 96.8%, respectively).

The mean age of the DOTP and placebo groups was 69.6 (range, 54–82) years and 69.5 (range, 51–81) years, respectively, whereas the BMI (range) was 23.7 (range, 18.4–30.0) kg/m^2^ and 22.8 (range, 16.9–34.5) kg/m^2^, respectively (Table 2). The Japanese Ministry of Health, Labor, and Welfare [10] lists the average BMI at 24.4 kg/m^2^ for males and 22.6 kg/m^2^ for females aged 40–69 years and 23.4 kg/m^2^ for males and 23.0 kg/m^2^ for females aged 70–84 years. Therefore, the participants’ average BMI was typical of the Japanese population at these ages.

### 3.2. Cognitive Function

Table 3 shows the pre- and post-trial cognitive function measurement outcomes and the results of repeated-measures ANCOVA. A significant time × group interaction was found in the complex attention domain (*p* = 0.049). In addition, a simple main effects analysis showed that DOTPs performed better than the placebo group in the post test (*p* < 0.001). By contrast, the other domains showed no significant time × group interactions.

The main effect of time was significant in all domains, except for composite memory (*p* = 0.212), verbal memory (*p* = 0.159), visual memory (*p* = 0.778), and simple attention (*p* = 0.191). In addition, the effect sizes of complex attention (d = 0.36), cognitive flexibility (d = 0.29), processing speed (d = 0.35), executive function (d = 0.36), and simple attention (d = 0.17) were largest in the DOTP group (Table 3).

## 4. Discussion

People in Japan have the longest life expectancy in the world [11]. In fact, by 2022, the population aged 65 and over will account for 29.1% of the total population. Japan is now already a “super-aging” society. Therefore, one of the primary goals of the Japanese government is to extend “healthy life expectancy”—that is, to maintain the health and well-being of Japan’s older adults. This situation has led to an increased public awareness of the importance of a healthy lifestyle. The drugs used for treatment of various chronic diseases are associated with numerous side-effects, which can lead to cognitive function deterioration in older adults. This warrants minimizing medications, which can adversely affect the quality of life in older adults. Therefore, the Japanese government has an important task to extend healthy life expectancy by consciously altering diet. Various health foods and supplements have been proposed to meet these goals [12,13]. Among them, the MedDiet has shown a possible preventive effect against various diseases [6,13]. The MedDiet, which is unfamiliar in Japan, is derived from the traditional and healthy eating patterns of the people of countries bordering the Mediterranean, such as Italy, Greece, and Spain. The active ingredients of olives, which are commonly consumed in the MedDiet, have beneficial effects on human health. Among various olive products, extra virgin olive oil (EVOO) has gained popularity due to its monounsaturated fatty acids and other trace constituents such as phenolic compounds that provide anti-inflammatory and antioxidant properties that may protect the body against diseases such as cancer, cardiovascular disease, and hypertension. Hence, exploring the MedDiet holds great potential for reducing chronic diseases and their risk factors that plague the older adults in Japan. They reduce the risk of cardiovascular disease and cancer and improve cognitive function [5,14].

Among the phenolic compounds in olives, hydroxytyrosol (HYD) has received particular attention. When consumed in the right quantity, HYD can extend healthy life expectancy and maintain or improve cognitive function in Alzheimer’s disease (AD).

The MedDiet’s most representative ingredient is olive, and its related products are olive oil (virgin olive oil) and DOTPs, both of which contain HYD. The antioxidant, anti-inflammatory, anti-atherogenic, and anti-thrombotic properties of HYD help maintain wellness [15,16]. Therefore, we focused on the effects of DOTPs containing HYD on the cognitive function of middle-aged and older adults.

Recently, the European Food Safety Authority (EFSA)’s comprehensive European food consumption database has reported the average HYD content in olives and olive oil as 0.6 and 0.005 mg/g, respectively [17]. In other words, the HYD contents (16.2 mg/g) in DOTPs used in the study (Table 1, Figure 2) are 26-fold and 3,115-fold higher in olives and olive oil than those reported by the EFSA, respectively. This means that the HYD content in DOTPs is higher than that in olive oil (including virgin olive oil).

In this study, all the participants had uniform characteristics, such as physical condition, education level, blood pressure, heart rate, and drug use; thus, there was no difference in the results of DOTP and placebo groups, which were influenced by these parameters (Table 2).

The participants in the DOTP group received HYD 32.4 mg/day (a total of approximately 2721.6 mg) over a 12-week period, which was 40.5–97.2 times higher than that received by the participants in the placebo group (0.2–0.8 mg/day, amounting to a total of approximately 33.6–67.2 mg over the 12-week period). The study focused on understanding the changes in cognitive function after consumption of a higher amount of HYD through DOTPs than that from consumption through a normal diet.

Table 3 shows the results of comparing various domains of cognitive function using Cognitrax pre- and post-test. The participants from the placebo group, who also received olive oil (0.1–0.4 mg/g HYD) twice daily for 12 weeks, showed favorable results in the cognitive domains for middle-aged and older adults. However, the participants from the DOTP group outperformed the placebo group in multiple cognitive domains.

Cognitrax is a standardized general assessment method involving computerized tests that assess multiple cognitive domains. Among the cognitive function items, complex attention (interaction: group × time) showed a significant difference between the two groups (*p* = 0.049). In addition, the simple main effect of the item significantly improved in the DOTP group (*p* < 0.001) compared with that in the placebo group (*p* = 0.575) before and after the intervention. Generally, humans show a decline in attentional efficiency with age [18]. However, the results of this study showed that middle-aged and older adults who consumed DOTPs for 12 weeks showed a valid change in complex attention. Thus, ingestion of DOTPs, which contains high concentrations of HYD, helped in maintaining or improving complex attention in older adults.

The time effect showed a significant difference (*p* < 0.05) in psychomotor speed, reaction time, cognitive flexibility, processing speed, executive function, and motor speed. Among these, motor speed showed an improvement in the placebo group. It was unclear whether this was due to the olive oil being mixed into the placebo when it was consumed. Olive oil also contains a small amount of HYD, which may be an indication of its positive effect.

Except for motor speed, the DOTP group showed more time-dependent changes in all the other mentioned cognitive functions than the placebo group. In other words, it was found that the intake of DOTPs with a high concentration of HYD caused beneficial changes in the pre-mentioned five cognitive function items. This result is congruent with those of previous studies showing that HYD in olives improves human cognitive function [19,20].

**Figure 3:**
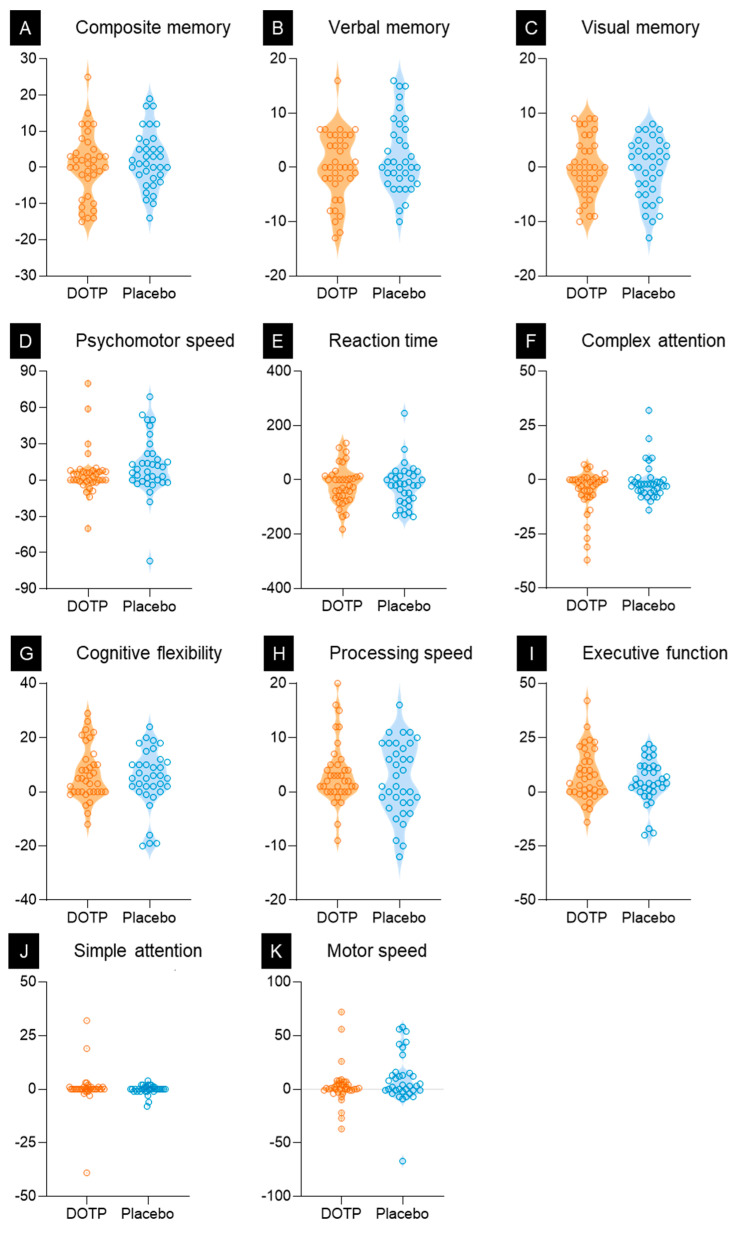
Individual change (empty circle) in the cognitive function score of middle-aged and older adults of the desert olive tree pearls (DOTPs) versus the placebo group at 12 weeks. The outcome of cognitive function using the Cognitrax test. Δ (post-test − pre-test).

As shown in Figure 3, the difference in cognitive function between the participants from DOTP and placebo groups was small, indicating that the placebo was also effective. Ingestion of high concentrations of HYD in DOTPs significantly improved the cognitive function of older adults compared to that of middle-aged adults (Table S1). This result indicates that DOTPs are more effective in improving the cognitive function of older adults (61–82 years) than that of middle-aged adults (51–60 years).

In middle-aged Japanese people, who consume less olive-related foods than Westerners owing to dietary differences, the 12-week dietary change had a positive effect on cognitive function. The results of this study confirmed that even at 12 weeks, the results were significant (*p* < 0.05) for cognitive functions of complex attention, reaction time, cognitive flexibility, processing speed, and executive function. Daily consumption of DOTPs, which contain high concentrations of HYD, has been suggested to have a beneficial effect on maintaining and improving cognitive function in Japanese elderly people who are unfamiliar with olive products. In other words, DOTP consumption may play a crucial role in extending healthy life expectancy, thereby alleviating problems faced by older adults Japan.

## 5. Conclusions

The benefits of the MedDiet to human health are well known. However, its benefits in the Asian population, who are unfamiliar with the MedDiet, is relatively unexplored. To the best of our knowledge, this is the first study to examine the effects of olives, a typical MedDiet ingredient, on cognitive function in the Japanese older adults. In particular, trying desert olive tree pearls (DOTPs), which are rich in hydroxytyrosol (HYD), which is known to have positive effects on cognitive function, was a challenge. The results of this study showed that HYD-rich DOTPs can improve cognitive function in Japanese middle-aged and older adults. In particular, there were significant effects on complex attention, psychomotor speed, reaction time, cognitive flexibility, processing speed, and executive function among the cognitive function items compared to the placebo group. These results suggest that DOTPs in the MedDiet, which is unfamiliar to Japanese middle-aged and older adults, may be a soft solution to the problems of the elderly in Japan. However, these changes were representative of our study, which was conducted over a short period of 12 weeks. Further long-term intervention studies are warranted to track changes in the cognitive function of this population.

## Figures and Tables

**Table 1:** Polyphenols in desert olive tree pearls.

Polyphenols	Content (mg/g)
Hydroxytyrosol	16.2
Tyrosol	2.8
Oleocantal	3.8
Oleacein	1.7
Total polyphenols	55.6

**Table 2:** Characteristics of participants by each group.

	All (*n* = 72)		DOTP (*n* = 36)		Placebo (*n* = 36)		*p*-Value
	Range	Mean (SD)	Range	Mean (SD)	Range	Mean (SD)	
Age (years) ^†^	51–82	69.5 (7.6)	54–82	69.6 (7.1)	51–81	69.5 (8.1)	0.939 ^†^
Height (cm) ^†^	137.7–179.1	156.4 (9.1)	139.5–179.1	156.8 (9.7)	137.7–176.8	156.0 (8.4)	0.716 ^†^
Body weight (kg) ^†^	39.7–94.6	57.1 (10.3)	39.7–76.8	58.4 (8.9)	40.7–94.6	55.6 (11.5)	0.252 ^†^
Body mass index (kg/m^2^) ^†^	16.9–34.5	23.2 (3.1)	18.4–30.0	23.7 (2.4)	16.9–34.5	22.8 (3.6)	0.208 ^†^
Education (years) ^†^							0.208 ^a^
Middle school	2 (2.8)		2 (5.5)		0 (0.0)		
High school	27 (37.5)		15 (41.7)		12 (33.3)		
University	43 (59.7)		19 (52.8)		24 (66.7)		
MMSE (points)	27–30	29.3 (0.9)	27–30	29.1 (1.0)	27–30	29.5 (0.9)	0.152 ^a^
Women (*n* (%)) ^a^	53		26.0		27		0.789 ^a^
Blood pressure (mmHg) ^†^	76–186	132.9 (19.8)	95–186	135.4 (19.4)	76–180	130.5 (20.2)	0.303 ^†^
Diastolic blood pressure (mmHg) ^†^	51–106	81.6 (12.8)	59–106	82.8 (12.7)	51–104	80.4 (13.0)	0.421 ^†^
Heart Rate (bpm) ^†^	54–104	74.1 (11.2)	54–104	74.8 (11.3)	54–104	73.3 (11.3)	0.574 ^†^
Take a Medicine							
Antihypertensive (*n* (%)) ^a^	21 (29.2)		11 (30.6)		10 (27.8)		0.795 ^a^
Diabetes (*n* (%)) ^a^	5 (6.9)		3 (8.3)		2 (5.6)		0.643 ^a^
Cerebrovascular heart disease (*n* (%)) ^a^	4 (5.6)		1 (2.8)		1 (2.8)		0.303 ^a^
Blood viscous reducer (*n* (%)) ^a^	3 (4.2)		1 (2.8)		2 (5.6)		0.394 ^a^
Physical function							
Grip strength (kg)	26.2 (6.4)		25.7 (6.6)		26.7 (6.3)		0.534
One-leg balance with eyes open (s)	49.2 (18.1)		50.7 (17.3)		47.7 (18.9)		0.439
Five-repetition sit-to-stand (s)	6.07 (1.51)		6.32 (1.81)		5.81 (1.10)		0.162
Timed up and go (s)	5.25 (0.82)		5.24 (0.83)		5.27 (0.81)		0.890

Note: ^†^ Each value is presented as mean (standard deviation). The *p*-value for differences between groups was calculated using analysis of variance for continuous variables and ^a^ χ^2^ tests for categorical variables. DOTP: desert olive tree pearl group, MMSE: Mini-Mental State Examination.

**Table 3:** Cognitive function by group at baseline and follow-up.

Variables	Unit	Group	*n*	Pre-Test Mean (SD)	Post-Test Mean (SD)	Effect Size (Cohen’s *d*)	Interaction (Groups × Time)	Simple-Main Effect	Group Effect	Time Effect
Composite memory	points	DOTP	34	90.7 (9.6)	91.2 (11.6)	0.05	0.455		0.376	0.212
		Placebo	33	92.3 (10.8)	94.6 (10.4)	0.22				
Verbal memory	points	DOTP	34	48.3 (6.2)	48.4 (7.5)	0.02	0.259		0.412	0.159
		Placebo	33	48.7 (7.5)	50.9 (6.8)	0.31				
Visual memory	points	DOTP	35	42.3 (5.1)	42.3 (5.7)	0.00	0.754		0.309	0.778
		Placebo	34	43.7 (5.6)	43.3 (5.2)	−0.07				
Psychomotor speed	points	DOTP	32	144.4 (31.6)	150.6 (28.3)	0.21	0.255		0.611	0.002
		Placebo	33	138.1 (33.6)	150.6 (31.6)	0.38				
Reaction time *	points	DOTP	34	834 (124.2)	815.7 (140.0)	0.12	0.857		0.072	0.040
		Placebo	33	779.0 (116.5)	759.0 (114.4)	0.17				
Complex attention *	points	DOTP	35	19.1 (17.7)	13.5 (15.8)	0.36	0.049	<0.001		
		Placebo	34	12.2 (8.6)	11.3 (13.2)	0.08		0.572		
Cognitive flexibility	points	DOTP	34	17.8 (24.7)	24.7 (22.3)	0.29	0.441		0.323	<0.001
		Placebo	34	24.5 (20.8)	29.4 (24.8)	0.21				
Processing speed	points	DOTP	35	46.9 (9.3)	50.4 (10.6)	0.35	0.542		0.331	<0.001
		Placebo	33	45.3 (12.1)	47.8 (12.6)	0.20				
Executive function	points	DOTP	3	18.7 (23.7)	26.7 (20.2)	0.36	0.248		0.309	<0.001
		Placebo	34	25.8 (20.6)	30.7 (23.4)	0.22				
Simple attention	points	DOTP	34	36.2 (10.6)	37.9 (8.7)	0.17	0.116		0.243	0.191
		Placebo	34	38.9 (1.2)	38.8 (2.3)	−0.06				
Motor speed	points	DOTP	32	96.4 (26.4)	99.7 (22.6)	0.13	0.217		0.763	0.018
		Placebo	33	91.6 (25.3)	101.6 (24.3)	0.40				

Note: Each value is presented as mean (standard deviation), covariate for educational years, *p* < 0.05, Cohen’s-d: |0.2 ≤ d< 0.5| = small, |0.5 ≤ d < 0.8| = moderate, |0.8 ≤ d| = large. * A lower score represents a better score. DOTP: desert olive tree pearl group.

## Data Availability

Not applicable.

## Supplementary Materials

The following supporting information can be downloaded at: https://www.mdpi.com/article/10.3390/nu15143234/s1, Table S1: Information on the amount of change and improvement rate of cognition function in this study.
