# Supplementary material for: Effects of Desert Olive Tree Pearls Containing High Hydroxytyrosol Concentrations on the Cognitive Functions of Middle-Aged and Older Adults
Source: Nutrients. 2023 Jul 21;15(14):3234. doi: 10.3390/nu15143234 (PMC10383185; doi:10.3390/nu15143234)
Supplement: Supplementary file 1 [file nutrients-15-03234-s001.zip › nutrients-2492335-supplementary.pdf]

Table S1. Information on the amount of change and improvement rate of cognition function in this study

		DOTP						Placebo					
	Unit	Age	n	Pre-test Mean (SD)	Post-test Mean (SD)	Amount of change (Post-Pre)	Improvement rate (%)	Age	n	Pre-test Mean (SD)	Post-test Mean (SD)	Amount of change (Post-Pre)	Improvement rate (%)
Blood test													
BDNF	pmol/L	54-63	7	501.5 (381.0)	336.0 (384.0)	-165.5	-33.0	51-64	8	488.7 (132.8)	224.3 (128.0)	-264.400	-54.103
		65-74	17	646.8 (258.5)	387.8 (214.3)	-259.0	-40.0	66-74	19	608.4 (220.3)	338.0 (300.0)	-270.400	-44.444
		75-82	12	511.3 (274.3)	336.7 (219.5)	-174.6	-34.1	75-81	9	604.3 (234.9)	408.1 (193.3)	-196.200	-32.467
Cognitive function (Cognitrax)													
Composite Memory	points	54-63	7	98.6 (10.8)	94.1 (15.1)	-4.5	-4.6	51-64	8	96.5 (6.6)	98.4 (8.4)	1.9	2.0
		65-74	17	89.1 (7.7)	91.6 (9.2)	2.5	2.8	66-74	19	91.9 (9.0)	94.5 (8.9)	2.6	2.8
		75-82	12	88.2 (9.4)	88.6 (12.8)	0.4	0.5	75-81	9	88.9 (16.4)	90.9 (14.6)	2.0	2.2
Verbal Memory	points	54-63	7	53.4 (6.3)	51.0 (9.6)	-2.4	-4.5	51-64	8	51.6 (4.8)	52.0 (5.2)	0.4	0.8
		65-74	17	47.0 (4.8)	49.6 (5.5)	2.6	5.5	66-74	19	47.8 (7.7)	51.4 (5.1)	3.6	7.5
		75-82	12	46.8 (6.8)	45.0 (8.1)	-1.8	-3.8	75-81	9	47.6 (9.4)	48.6 (10.8)	1.0	2.1
Visual Memory	points	54-63	7	45.1 (6.0)	43.1 (6.5)	-2.0	-4.4	51-64	8	44.9 (5.4)	46.4 (5.0)	1.5	3.3
		65-74	17	42.1 (4.8)	42.0 (4.3)	-0.1	-0.2	66-74	19	44.3 (3.9)	42.4 (4.3)	-1.9	-4.3
		75-82	12	40.8 (4.8)	42.1 (7.1)	1.3	3.2	75-81	9	41.3 (8.3)	42.3 (6.6)	1.0	2.4
psychomotor Speed	points	54-63	7	141.6 (44.5)	156.4 (25.1)	14.8	10.5	51-64	8	162.4 (16.9)	177.1 (13.2)	14.7	9.1
		65-74	17	144.9 (34.1)	150.3 (35.7)	5.4	3.7	66-74	19	137.2 (33.3)	149.6 (34.2)	12.4	9.0
		75-82	12	145.7 (17.4)	146.9 (17.8)	1.2	0.8	75-81	9	115.6 (32.7)	126.3 (15.7)	10.7	9.3
Reaction Time※	points	54-63	7	753.1 (73.6)	712.9 (75.4)	-40.2	5.3	51-64	8	704.8 (113.3)	673.9(78.3)	-30.9	4.4
		65-74	17	832.2 (142.6)	820.4 (143.5)	-11.8	1.4	66-74	19	798.5 (100.9)	779.1 (120.5)	-19.4	2.4
		75-82	12	893.6 (88.1)	879.9 (136.5)	-13.7	1.5	75-81	9	813.6 (136.7)	804.6 (90.6)	-9.0	1.1
Complex Attention※	points	54-63	7	10.9 (9.9)	8.3 (11.3)	-2.6	23.9	51-64	8	8.0 (4.4)	6.3 (5.4)	-1.7	21.3
		65-74	17	18.4 (15.0)	12.1 (7.8)	-6.3	34.2	66-74	19	10.8 (5.4)	8.6 (8.0)	-2.2	20.4
		75-82	12	26.2 (23.6)	19.5 (24.7)	-6.7	25.6	75-81	9	20.7 (13.5)	24.4 (21.6)	3.7	-17.9
Cognitive Flexibility	points	54-63	7	26.1 (22.6)	33.6 (23.2)	7.5	28.7	51-64	8	38.9 (12.9)	43.6 (11.3)	4.7	12.1
		65-74	17	18.6 (26.1)	24.9 (19.0)	6.3	33.9	66-74	19	27.3 (12.9)	35.1 (12.8)	7.8	28.6
		75-82	12	13.9 (24.4)	22.6 (23.9)	8.7	62.6	75-81	9	3.8 (26.9)	2.4 (35.1)	-1.4	-36.8
Processing Speed	points	54-63	7	52.1 (10.6)	55.7 (15.3)	3.6	6.9	51-64	8	53.7 (9.6)	59.4 (9.2)	5.7	10.6
		65-74	17	47.5 (7.8)	50.7 (8.8)	3.2	6.7	66-74	19	46.5 (10.0)	49.6 (8.3)	3.1	6.7
		75-82	12	44.0 (9.0)	46.4 (9.6)	2.4	5.5	75-81	9	35.3 (12.7)	33.6 (10.8)	-1.7	-4.8
Executive Function	points	54-63	7	26.7 (22.1)	34.6 (21.5)	7.9	29.6	51-64	8	40.3 (12.7)	44.3 (11.5)	4.0	9.9
		65-74	17	18.0 (26.2)	27.1 (17.5)	9.1	50.6	66-74	19	28.4 (12.7)	35.7 (13.0)	7.3	25.7
		75-82	12	13.9 (23.0)	21.5 (23.4)	7.6	54.7	75-81	9	5.6 (26.8)	5.9 (32.6)	0.3	5.4
Simple Attention	points	54-63	7	39.0 (0.8)	39.4 (1.0)	0.4	1.0	51-64	8	39.0 (1.1)	38.9 (1.6)	-0.1	-0.3
		65-74	17	38.1 (5.2)	39.5 (0.9)	1.4	3.7	66-74	19	39.3 (1.0)	39.6 (0.6)	0.3	0.8
		75-82	12	31.6 (17.2)	34.6 (15.1)	3.0	9.5	75-81	9	38.0 (1.3)	36.6 (4.3)	-1.4	-3.7
Motor Speed	points	54-63	7	88.9(37.6)	100.0 (16.8)	11.1	12.5	51-64	8	107.3 (16.0)	117.8 (11.1)	10.5	9.8
		65-74	17	96.4 (28.6)	98.1 (30.3)	1.7	1.8	66-74	19	90.1 (27.8)	98.9 (30.0)	8.8	9.8
		75-82	12	101.7 (10.6)	101.8 (11.4)	0.1	0.1	75-81	9	79.4 (21.4)	91.4 (8.9)	12.0	15.1

Note: DOTP; desert olive tree pearls, †Each value is presented as mean (standard Deviation), ※ Lower values represent better score.
